# Psychological Flexibility and Inflexibility of University Students: An In-Depth Qualitative Study

**DOI:** 10.3390/ijerph22071141

**Published:** 2025-07-18

**Authors:** Wendy Cervantes-Perea, Jone Martínez-Bacaicoa, Manuel Gámez-Guadix

**Affiliations:** 1 Faculty of Health Sciences, Universidad del Magdalena, Santa Marta 470004, Colombia; 2 Faculty of Psychology, Autonomous University of Madrid, 28049 Madrid, Spain; jone.martinez@uam.es

**Keywords:** psychological flexibility, psychological inflexibility, university students

## Abstract

In the Hexaflex model of Acceptance and Commitment Therapy (ACT), psychological flexibility refers to the ability to openly embrace difficult thoughts and emotions while acting in alignment with personal values. In contrast, psychological inflexibility involves rigid avoidance and control strategies that hinder adaptive functioning. Although previously studied, more culturally relevant evidence is needed to inform interventions that promote well-being and mental health among Latin American students. This study explored manifestations of psychological flexibility and inflexibility in 15 undergraduate students from the University of Magdalena in Colombia (mean age = 20.13 years; 53.33% female) through semi-structured, face-to-face interviews (~45 min each). Data were analyzed using Interpretative Phenomenological Analysis (IPA), focusing on how participants described and made sense of their experiences. A total of 25 emergent themes were identified and grouped into 12 subordinate themes, mapped onto the 6 core ACT processes. The participants reported efforts to control or avoid distressing internal experiences, often resulting in difficulty acting in accordance with their values. The findings highlight a recurring ambivalence between avoidance and acceptance, and barriers to committed action, underscoring the dynamic interplay between flexibility and inflexibility. These results support the relevance of ACT-based interventions, such as structured group sessions that foster acceptance, mindfulness, and values-based behavior. Integrating this training into counseling and academic support services could enhance students’ well-being and performance. Future research should examine these dynamics longitudinally and across diverse contexts.

## 1. Introduction

Psychological flexibility is one of the central concepts of Acceptance and Commitment Therapy (ACT). ACT is a therapeutic model aimed at modifying the relationship individuals have with their thoughts and the influence these exert on their behavior so that the latter is guided by personal values rather than relational frames and verbal rules that are shaped through language [1]. Being psychologically flexible involves opening up to discomfort as an inevitable part of life while moving toward what is truly valuable [2] by setting aside internal struggles to focus on the here and now and persisting in or consistently adjusting one’s behavior so that it aligns with one’s chosen values.

Accordingly, human behavior is analyzed in relation to the context in which it occurs. The therapeutic approach of ACT is supported by the experimental analysis of language and human cognition that was developed extensively through relational frame theory. This theory highlights how language establishes connections that shape individuals’ perceptions and interactions with the world [3,4].

The repertoire of behaviors that individuals learn and maintain often constitutes the means by which they evaluate, judge, and form opinions about their feelings and thoughts—referred to in ACT as private events—without necessarily representing the reality of their experiences accurately. Wilson and Luciano emphasized that the relationship between private events and actions is influenced by arbitrary interactions between personal history and verbal context, which results from numerous perspective frames converging in the emergence of self-dimensions [1]. In this framework, the Hexaflex model synthesizes the patterns through which private events affect behavior, encompassing six key processes that regulate adaptive or rigid responding.

These processes are described as experiential acceptance versus avoidance, defusion versus fusion, self-as-context versus self-as-content, present-moment awareness versus lack of present-moment awareness, values clarification versus lack of values clarification, and committed action versus inaction [5]. Acceptance involves embracing internal experiences without unnecessary struggle, while experiential avoidance reflects efforts to suppress discomfort, often at a psychological cost [6,7]. Defusion enables individuals to distance themselves from unhelpful thoughts, whereas cognitive fusion rigidly links actions to literal interpretations of thoughts [8,9,10]. Self-as-context promotes observing oneself from a broader perspective, while self-as-content restricts identity to self-evaluative thoughts [11,12,13]. Present-moment awareness supports attention and emotional regulation, in contrast to mind wandering and disconnection [6,8,14]. Clarification of values provides direction [8], and committed action translates this clarity into consistent, goal-oriented behavior, as opposed to inaction driven by avoidance and fear of failure [6,15,16].

Understanding these six processes is valuable not only because they help explain how individuals respond to challenges [17] but also because they are closely linked to psychological well-being. Specifically, psychological flexibility has been associated with lower stress, depression, and anxiety, whereas inflexibility strongly predicts worse mental health outcomes [18,19,20]. In fact, a growing body of research, including a recent meta-analysis, has examined the psychological well-being of this population, confirming that interventions aimed at enhancing psychological flexibility—such as ACT—are effective in reducing distress and fostering resilience among college students [19,20,21,22]. Therefore, psychological flexibility may be especially relevant for populations exposed to high levels of stress. University students represent one such group, as they often navigate demanding academic environments and are recognized as being vulnerable to mental health difficulties [23].

Research in Latin America has confirmed this issue. In a study in Peru, 40.3% of students exhibited medium to high levels of experiential avoidance, with a higher incidence among those in the early stages of adulthood (18–25 years old) [24]. In Brazil, researchers found that 39.9% of students showed symptoms of anxiety, depression, and somatization [25], while a study involving nursing students showed that more than a third suffered from depressive disorders, anxiety, and somatoform symptoms [26]. In a study conducted in Chile, 29% of university students exhibited depression, 53.2% experienced anxiety, and 47.8% reported stress [27], all of which negatively impacted their well-being and academic performance. 

Taken together, these studies reveal that symptomatology associated with psychological disorders is experienced by students in response to the demands of the university environment [28]. Concern about potential failure as well as self-perceptions of being incapable of managing these demands may facilitate the emergence or persistence of control strategies aimed at avoiding exposure to stimuli with aversive meanings or engagement in a relentless search for explanations to understand the perceived distress. When distress is interpreted through such strategies, patterns of psychological inflexibility are activated. These may promote a perpetual and futile struggle to escape suffering, which may prove to be deeply harmful and disabling in daily life [8].

These findings underscore the need for further research to deepen the understanding of how personal psychological resources can facilitate or hinder students’ adaptation to the demands of university life. However, despite significant theoretical advances in the understanding of psychological flexibility, there remains a critical gap in the qualitative literature regarding how these processes are subjectively experienced and interpreted by university students in Latin American contexts. While quantitative studies have examined the links between psychological flexibility, coping strategies, emotional regulation, and academic performance [28,29], few qualitative investigations have explored how flexibility and inflexibility actually manifest in students’ daily experiences and how these meanings shape their behavior. Furthermore, there is limited exploration of the theoretical, methodological, and practical challenges involved in cultivating psychological flexibility in educational settings.

Addressing this gap is essential, as psychological flexibility enables students to cope more adaptively with academic stressors and interpersonal challenges, whereas inflexibility can amplify avoidance, rumination, and disengagement, increasing vulnerability to mental health difficulties and poor academic outcomes [6,30]. Therefore, this study aims to qualitatively explore how psychological flexibility and inflexibility manifest among Latin American university students and how these patterns are associated with their coping and adjustment. It is anticipated that students exhibiting greater inflexibility will report more frequent use of avoidance strategies and less effective emotion regulation. Practically, these insights can inform the design of culturally sensitive psychoeducational programs that foster psychological flexibility and promote mental health and academic success in higher education.

## 2. Materials and Methods

### 2.1. Design

This study employed Interpretative Phenomenological Analysis (IPA) as the guiding qualitative approach. IPA is firmly grounded in phenomenology, hermeneutics, and idiography, and focuses on exploring how individuals make sense of their lived experiences [31]. Unlike generic thematic analysis, IPA integrates thematic thinking within an interpretative and idiographic framework, emphasizing detailed examination and double hermeneutics. Although thematic patterns are identified, they are always contextualized within participants’ meaning-making, rather than treated as abstract categories [32]. In this study, IPA was used to elicit rich, first-person accounts of students’ psychological flexibility and inflexibility, providing insights into how these processes are experienced and interpreted in the context of higher education.

### 2.2. Participants

Purposive sampling was used to recruit 15 undergraduate students (eight women and seven men; mean age = 20.13 years, *SD* = 1.30) from various academic programs at the University of Magdalena in northern Colombia. Table 1 summarizes the main demographic characteristics of the participants.

The participants were selected based on the following inclusion criteria: (1) being 18 years or older, (2) being enrolled as an undergraduate student at the University of Magdalena, (3) having completed at least one semester, and (4) being willing to share personal experiences related to psychological flexibility and inflexibility. No specific exclusion criteria were applied other than unwillingness to participate. The sample size of 15 participants was determined following established guidelines for achieving sufficient depth in idiographic qualitative research and was confirmed by thematic saturation, which was monitored throughout the analysis; no new themes emerged after the twelfth interview. The participants represented various academic years, from first to fourth year. Although individual socioeconomic status was not directly measured, all participants came from similar middle-income backgrounds typical of the university’s student population.

### 2.3. Qualitative Data Analysis 

All interviews were conducted face-to-face by the first author, who is trained in qualitative interviews. Each interview lasted approximately 45 min and was recorded with the informed consent of the participants. A script based on the processes of psychological flexibility and inflexibility [5] and the data collection principles of IPA was used during the interviews (see Appendix A). All interviews were conducted in Spanish and later transcribed for analysis.

The questions were neutral, open-ended, and flexible, which allowed the interviewer to explore emerging topics [32]. Throughout this study, a reflective journal was maintained to document interviewer positionality, assumptions, and potential biases, and to ensure transparency in the researcher–participant relationship.

### 2.4. Data Analysis

The interviews were audio-recorded, transcribed verbatim, and analyzed following the classic six-step IPA [32,33]; a summary of the steps is presented in Table 2.

The research team deliberately chose not to use qualitative data analysis software in order to engage more fully and reflexively with the participants’ narratives. This manual approach was grounded in a commitment to deep, conscious immersion in the data, which allowed the researchers to attend closely to the experiential, linguistic, and contextual subtleties of the accounts. The analysis process included multiple readings of the transcripts, systematic annotation, identification and refinement of emergent themes, and cross-case examination. All interpretive decisions were discussed collaboratively within the research team to enhance transparency, analytical rigor, and trustworthiness.

Although thematic thinking was employed to organize patterns, it remained embedded within the epistemological and interpretative boundaries of IPA, preserving the idiographic focus and the double hermeneutic process. Peer debriefing among team members was conducted to review coding and theme development, and participant validation (member checking) was carried out by sharing thematic summaries with a subset of participants to ensure interpretive accuracy. Triangulation was supported through comparison across cases and researcher reflections. Data saturation was confirmed when no new themes emerged in later interviews.

## 3. Results

As a first step, this study explored whether participants had any prior knowledge of psychological flexibility, with the aim of eliciting spontaneous and unbiased responses. The first two interview questions focused on whether participants were familiar with the concept or had ever heard of behaviors associated with psychologically flexible individuals. None of the participants reported prior familiarity with the concept, stating they had never read about it or heard about it before.

However, regarding the behaviors and attitudes that participants believed should characterize a psychologically flexible person, a variety of responses were obtained. Similarities across these responses were identified, which allowed us to group and label the attributes. Consequently, categories were established based on the participants’ perspectives. The categories open and reflective mindset, emotional management, and openness to change referred to behaviors directed toward oneself, while the social skills category was more oriented toward behaviors directed toward others.

Some responses from the open and reflective mindset category included:

*It would be a very open-minded person, very spontaneous with ideas. I’m not saying they are entirely positive, but they don’t see many obstacles; they always think about moving forward.* 

(SP1 Male, 22 years old)

*They are a person who doesn’t cling to a single concept of things. I mean, even if things are stipulated to be a certain way, that person can change things and make them better.* 

(SP6 Male, 22 years old)

These extracts suggest that, for these students, an open and reflective mindset entails a dynamic balance between spontaneity and adaptability. Notably, this openness is not perceived as passive acceptance but rather as an active capacity to reframe challenges as opportunities for personal growth and contextual improvement.

In the emotional management category, the participants’ responses included:

*They wouldn’t repress themselves; they would let themselves be carried by who they are, what they feel, [and] the emotions they have in the moment.* 

(SP2 Male, 20 years old)

*They are a person who has control over what they feel because they are capable of observing their surroundings.* 

(SP9 Female, 21 years old)

Similarly, when discussing emotional management, the participants highlighted that effective regulation is intertwined with an attitude of mindful self-observation rather than rigid suppression.

The openness to change category emerged from responses such as:

*[They are] able to face different situations, I don’t know how to explain it, but they would help themselves move forward.* 

(SP3 Female, 20 years old)

*[They are] able to solve problems, [get] through them successfully. At first, I perceived it as a weakness, but I believe it’s more about having the ability to solve [problems].* 

(SP14 Male, 18 years old)

These accounts suggest that, for these students, openness to change entails an active willingness to engage with uncertainty and a pragmatic orientation towards resolving obstacles.

The focus of the social skills category was attributes oriented toward others, for example:

*They must be … understanding … toward others; they must be sociable.* 

(SP11 Female, 19 years old)

*They must be a tolerant, respectful person and, above all, someone who has the ability to truly listen, not just hear, but [to] listen and understand what the other person is expressing.* 

(SP13 Female, 21 years old)

For these participants, social skills are not merely basic interactional competencies but are deeply embedded in relational sensitivity and ethical understanding.

The analysis of the interviews led to the identification of 25 emerging themes, which were grouped into 12 superordinate themes that corresponded to the 6 processes of psychological flexibility and inflexibility proposed in the Hexaflex Model of ACT.

The thematic organization outlined in Table 3 provides an integrated overview that bridges the raw experiential data with the conceptual framework of the Hexaflex Model. This overview serves as a roadmap for the detailed narrative and interpretative commentary that follows, wherein each theme is unpacked and illustrated with participants’ lived accounts.

### 3.1. Description of Results by Superordinate Theme

Following the thematic table, the results are presented in detail according to the superordinate themes and their associated emergent themes. These themes reflect how participants manifested psychological flexibility and inflexibility in their lived experiences.

#### 3.1.1. Manifestations Associated with the Control of Private Events

The experience of private events among the interviewed university students was characterized by a tendency to control the thoughts, emotions, and sensations they perceived as aversive. This manifested through deliberate strategies—either by clinging to mental content or by avoiding and invalidating such experiences.

One of the most common manifestations was rumination, in which the students focused on distressing thoughts or emotions, constantly reviewed them, and acknowledged their difficulty in detaching from them. Some of their statements reflected this recurrence:

*I think about it daily, and daily means out of 24 h a day, about 15 or 16 h at a minimum. I really think about it very often.* 

(SP1 Male, 22 years old)

*Everything I think about revolves around that, then the thoughts come that I’m useless, I mean, I start thinking about all the bad things.* 

(PS2 Male, 20 years old)

*So, if something bad happens to me, I start thinking about it, and I can’t concentrate on anything else. I mean, all my thoughts revolve around that. It’s like I have to resolve that situation to be able to continue with my day.* 

(SP12 Male, 22 years old)

One approach that the students resorted to when facing negative thoughts and uncomfortable emotions was to search for reasons to explain why the emotional experience was happening:

*When something happens to me, I spend a lot of time thinking about it, and all the time in my head it’s like why, why, why? I try to find an explanation or justification for what’s happening to me.* 

(SP3 Female, 20 years old)

*I always try to find an explanation for the things that happen to me; I don’t like to be left with doubts or with the questions that I impose on myself.* 

(SP7 Male, 19 years old)

For other participants, minimizing or suppressing their emotional reactions was a common strategy to manage internal discomfort. The most explicit form of this strategy was redirecting their attention to other activities, such as listening to music, exercising, or dancing. The repertoire was quite broad:

*So I channel my anger into training, lifting a lot of weight, like all my anger, all my rage, that strength, and being mentally exhausted.* 

(SP1 Male, 22 years old)

*I mainly tend to invalidate everything—any situation that generates anger or disappointment in me, I just try not to think about it for as long as possible.* 

(SP4 Female, 19 years old)

The invalidation of private events also manifested through behavior patterns related to avoidance, fleeing from exposure, and confrontation. Distancing and withdrawing from others was perceived by participants as a protective strategy to reduce emotional exposure and perceived interpersonal risk. A group of participants explained what this experience was like:

*I change my attitude; if I’m angry or sad, I distance myself, I shut down and don’t talk about it. So I carry that discomfort with me because I feel that if I am angry, I could offend or hurt someone.* 

(SP3 Female, 20 years old)

*I distance myself. I withdraw from everything and listen to music. I don’t have many friends to lean on, so it’s just me … and I cry a lot.* 

(SP7 Male, 19 years old)

*I isolate myself; I prefer to be alone.* 

(SP8 Female, 21 years old)

Overall, the participants described a marked tendency to control or avoid their internal experiences—particularly those perceived as distressing—through strategies such as rumination, overthinking, distraction, and withdrawal. Although these behaviors provided temporary relief, they reflect experiential avoidance, a central process in psychological inflexibility. From the ACT perspective, such avoidance limits emotional openness and undermines the development of adaptive coping in the long term.

#### 3.1.2. Manifestations Associated with the Acceptance of Private Events

Overall, the interviewed students did not describe strategies aimed at accepting or validating their internal experiences, nor did they report voluntarily allowing thoughts and emotions to arise without resistance. However, one participant acknowledged that acceptance involved a conscious decision to let go of what could not be controlled in order to restore psychological balance:

*If I see that I can’t solve it, then I say, why stress about it if I can’t fix it anyway? So I let it go, release it, and move on. Honestly, one feels lighter.* 

(SP6 Male, 22 years old)

Only isolated narratives revealed moments of acceptance in which participants allowed themselves to experience emotional discomfort without resorting to suppression or control. These rare instances underscore the challenge of cultivating openness to internal events, particularly in the absence of structured psychological training or therapeutic support. Within these accounts, acceptance appeared less as an intentional stance of mindful awareness and more as a functional strategy to regain stability in the face of unresolved emotional distress.

#### 3.1.3. Manifestations Associated with Observing Oneself Through the Content of Thoughts

The interviewed university students frequently exhibited a tendency to observe, define, and behave in alignment with their self-referential thoughts and beliefs. Three specific forms of this phenomenon were identified. First, several participants expressed self-criticism rooted in how they interpreted their own thoughts or emotions.

*I know I have anger issues that I can control. I try not to get angry, instead I try to ignore those times when many bad things or family problems occur. I try not to get involved because I feel I have a lot of anger issues.* 

(SP1 Male, 22 years old)

*I feel that my way of being sometimes gives the impression that I want to be liked by everyone, and I’ve received comments like “You want to please everyone.” I don’t know how I do that or if I do it unconsciously, but I tend to be that way, and then I feel bad because I don’t like that [other people] think that.* 

(SP3 Female, 20 years old)

A second pattern involved attributing negative meanings to the self-based on emotional states or cognitive content:

*Being a perfectionist really bothers me; that part of me causes a lot of stress because not everything can be perfect.* 

(SP11 Female, 19 years old)

*Many times, I stop doing things because I know I won’t feel good or because I believe I won’t do them well, that they won’t turn out right.* 

(SP14 Male, 18 years old)

Finally, many participants displayed signs of cognitive fusion, wherein identity was conflated with internal experiences—interpreting thoughts and emotions as intrinsic attributes of the self:

*I’m a very insecure person; I don’t like taking many risks. I always try to stay in a safe place.* 

(SP4 Female, 19 years old)

*I’m a bit manipulative, not in a bad way, but to achieve my goals. I’m always thinking about what benefits me and what doesn’t.* 

(SP7 Male, 19 years old)

These narratives revealed a consistent pattern of fusion between self-perception and mental content, in which negative thoughts and emotions became defining features of identity. This self-as-content perspective often led to self-criticism and rigid self-definitions, ultimately constraining behavioral flexibility. According to ACT, such fusion compromises the individual’s capacity to observe their internal experiences from a broader and more stable sense of self.

#### 3.1.4. Manifestations Associated with Observing Oneself Without Judgment

Only a few participants demonstrated the capacity to sustain a perspective beyond transient negative thoughts and emotions, maintaining a stable and balanced view of themselves despite contextual fluctuations. At least three participants articulated aspects of what they perceived to be their core essence traits considered stable across time and situations:

*Before, I felt like a fearful person, somewhat insufficient [and] incompetent, [but] now I feel like a person with many positive qualities. I’m still afraid of many things—that hasn’t changed—I’m still insecure, but now I move forward even with fear.* 

(SP2 Male, 20 years old)

*I am a sensitive person; that’s my essence, and I feel like I will never lose it. But perhaps before, I was the kind of sensitive person who allowed everything because I loved others.* 

(SP13 Female, 21 years old)

*When you’re a teenager, you see yourself one way, but when I entered university, I still thought the same things about myself, but my way of seeing myself to take risks and make decisions has changed a lot.* 

(SP14 Male, 18 years old)

These accounts suggest moments of connection with a more stable and accepting sense of self resembling the ACT construct of the observing self, which facilitates psychological flexibility by enabling individuals to witness their internal experiences without over-identifying with them.

#### 3.1.5. Manifestations Associated with Disconnection from the Present Moment

Strategies for controlling internal experiences employed by most of the students who participated in this study included being immersed in daily routines and constantly responding to everyday tasks without pause. This process encompassed a range of behaviors associated with avoiding or withdrawing not only from painful thoughts or emotions but also from the experience of living and interacting with others.

We therefore asked the participants: “*Can you explain to me what it’s like to live on autopilot?*” They all describe it as doing the same thing every day without much enjoyment. One participant described it as an itinerary that is followed day after day:

*Before going to bed, I start thinking: Tomorrow you have to get up at such and such a time, make breakfast at such and such a time, go to classes, then go to the gym, and when you get back from the gym, you have to do this and that, and so on every day.* 

(SP7 Male, 19 years old)

A significant proportion of the participants acknowledged the functionality of being focused on daily tasks and commitments, as this kept them away from private experiences, as evidenced by the following:

*I have so many things to do that I have no time for anything else, and it works for me because living like this helps me not think and not give myself space to feel bad.* 

(SP10 Female, 19 years old)

*What has worked for me to avoid feeling bad is doing lots of things. So, besides my academic workload, I’m also taking two more courses, and I really just get home exhausted and go to sleep.* 

(SP7 Male, 19 years old)

A group of students were aware that they had been living this way for quite some time; however, at the time of the interview, they had not taken any action to break out of the routine or monotony. For example, one student said:

*Because I’m even aware of it, but I don’t do anything to change it. It’s like I’m comfortable living this way, without anything out of the ordinary happening.* 

(SP11 Female, 19 years old)

In contrast, another group of students became aware during the interview itself that they were living this way:

*Right now, I’m realizing that I have been living like this. Until now, I hadn’t stopped to think about it; I saw it as normal, like everyone lives this way.* 

(SP3 Female, 20 years old)

*Just now I’m becoming aware of it; I hadn’t thought of it like this before. Until this moment, I saw it as part of my organization and wasn’t aware of how rigid it can sound or seem.* 

(SP7 Male, 19 years old)

From an interpretative phenomenological perspective, this set of narratives reveals an embodied experience of psychological disconnection that is normalized through routine and overactivity. Students appear to adopt busyness as a coping mechanism, which, while effective in numbing emotional discomfort, simultaneously distances them from present-moment awareness and authentic self-reflection. The absence of conscious contact with their internal world suggests a reduced capacity for mindful engagement and raises important questions about how emotional avoidance becomes embedded in the everyday life of emerging adults.

#### 3.1.6. Manifestations Associated with the Connection of the Present Moment

Instances of conscious engagement with present-moment experiences were relatively rare among the interviewed students. A few participants described routines that, while structured, were lived with awareness and a sense of enjoyment:

*I have a routine, but I don’t get lost in it; I mean, I enjoy my day.* 

(SP6 Male, 22 years old)

*I haven’t fallen into that state of doing things mechanically. I get up at 5, go to university, have lunch, go back to university, and then return home, but I am aware of my routine and try to do other things I enjoy.* 

(SP14 Male, 18 years old)

One participant explained what it meant to her to be in contact with the present moment:

*I have always felt that I pay a lot of attention to the things I do and that I enjoy them. I can be very busy, but even so, if something comes up and I can make time for it, I do—like going to chat with friends or getting some ice cream.* 

(SP15 Female, 19 years old)

These narratives suggest that connection to the present moment, although infrequent, is experienced as an active choice rather than a predetermined state. The participants who described such connection highlighted the intentionality of attending to everyday experiences with openness and flexibility. These accounts reveal evidence of psychological flexibility, where routine is not synonymous with disconnection, but rather allows for moments of spontaneity, enjoyment, and meaningful social interaction. Such moments may reflect an emerging capacity to step outside of automatism and connect with life as it unfolds.

#### 3.1.7. Manifestations Associated with Fusing with Thought Content

Cognitive fusion is the inability to differentiate thought content from the sense of self, to place what is happening in perspective, and to distance oneself from one’s mental content without being influenced by or hooked into emotions, memories, beliefs, or ideas. 

The participants’ narratives in response to the question “*Has it ever happened that you created a movie in your head that only existed there?*” revealed a tendency to fuse with their beliefs and thoughts, to become entangled with their distressing thoughts or feelings by applying various control mechanisms, which granted them the status of truth and considerably interfered with their circumstances and relationships with their environment. Examples of this were reported by these participants:

*I have only had one relationship, and there was a time when I would make up movies in my head, thinking he was seeing someone else: They would leave at the same time, so they must be together. I spent all my time thinking about that. I got obsessed. I was always angry because I assumed something was happening, and eventually, my relationship ended because of that.* 

(SP7 Male, 19 years old)

*I have a kind of multiverse of what could happen; I always see all the things that could happen, especially the worse-case scenarios, and I act accordingly.* 

(SP9 Female, 21 years old)

Another illustration of the interference caused by cognitive fusion interacting with context was noted by these participants:

*When I confront people, I’m often surprised because I take what I think as a given and feel entitled to confront them, and sometimes I go too far with what I say.* 

(SP12 Male, 22 years old)

*For example, sometimes I meet someone and feel like they judge me badly, so I avoid interacting with that person because of how they look at me.* 

(SP14 Male, 18 years old)

Regarding the possibility of being a spectator of their private events, most of the participants emphasized the difficulty of putting things into perspective and distancing themselves from their mental content. This was reflected in their responses to the question “Have you ever felt like a spectator of that movie?”

*No, I was the writer, director, producer, and protagonist but never the spectator.* 

(SP7 Male, 19 years old)

*No, during all that time I was completely immersed in the movie; that was my reality, and I didn’t allow myself to see any other reality.* 

(SP8 Female, 21 years old)

Another form of interference that disrupted the participants’ behavior was the tendency to fuse with past emotions that were negative, as expressed by these students:

*I haven’t had a romantic relationship in a long time. But I feel like I can’t feel anything for anyone. Since my uncle died, I haven’t been able to feel anything for anyone.* 

(SP1 Male, 22 years old)

*I carry that feeling of being watched, like it was throughout high school. Of not being able to do things because I know my parents wouldn’t like it, and in the end, I always end up feeling that way and prefer to do nothing.* 

(SP4 Female, 19 years old)

These findings point to a profound entrenchment in internal narratives, where participants did not merely report thoughts, they lived them as unquestionable truths. Rather than observing their mental content from a distance, the students described immersive experiences that shaped their emotional states, relationships, and daily behavior. Such narratives reveal the difficulty in adopting a metacognitive or observer stance, central to psychological flexibility. Within the IPA framework, this suggests that fusion not only distorts present-moment awareness but also narrows the repertoire of behavioral responses, reinforcing rigid patterns of self-definition and emotional avoidance. Cultivating defusion skills may be essential for promoting psychological adaptability in contexts marked by cognitive entanglement.

#### 3.1.8. Manifestations Associated with Separating from Thought Content

The ability to differentiate oneself from one’s private experiences was recognized by a participant, who noted the following:

*I don’t like to speculate because humans speculate for the worst, never for the best. And that leads to distress, misunderstandings, and problems.* 

(SP13 Female, 21 years old)

This same participant explained what it felt like to be a spectator of her life:

*At some point, I also learned to be a spectator, especially when I realized that [a] situation was beyond my control. So, I just let it unfold however it needed to because you can’t control everything; it’s exhausting, and I won’t do anything to change it.* 

One participant described it in terms of the need to distance oneself from thoughts to stop suffering:

*Sometimes you have to because if you can’t solve things, why live miserably? That way, you put an end to the mortification.* 

(SP6 Male, 22 years old)

When asked “*And when you see yourself as a spectator, what do you feel, what happens to you?*”, both participants noted how it freed them of the burden of carrying mental content:

*Calm, because I feel like I have let go, like I have taken a weight off my shoulders. It’s better to just let things happen.* 

(SP6 Male, 22 years old)

*It’s about accepting that you don’t have control over everything. It’s like taking a weight off your shoulders. It’s like saying, everything passes, and this will pass too, and everything will fall into place.* 

(SP13 Female, 21 years old)

These accounts illuminate a nascent capacity for defusion, wherein participants describe moments of stepping back from their thoughts and recognizing them as transient mental events rather than absolute truths. Such distancing enabled emotional relief and a diminished burden of control. While infrequent, these experiences suggest the potential for developing a more flexible perspective on internal experiences. Within the interpretative phenomenological framework, these insights reflect emerging processes of self-as-context that support psychological flexibility and promote adaptive functioning in emotionally challenging contexts.

#### 3.1.9. Manifestations Associated with Interference in Contacting Personal Values

For some of the participants, it was difficult to connect with what was truly important to them. Although they demonstrated the ability to identify and list what gave meaning to their lives, they encountered obstacles in paying attention to their values.

When asked “*What things are important to you?*,” all the participants responded immediately, without taking time to think, which demonstrated clarity about what gave their lives meaning and purpose. Their answers made it clear that the participants assigned value to family, well-being, health, relationships, professional development, and autonomy.

Some responses regarding family were as follows:

*The most important thing in my life is my grandmother. Obviously my parents too, but my grandmother and the two girls my uncle left behind are my greatest drive, one hundred percent.* 

(SP1 Male, 22 years old)

*My family. I think everything revolves around them. Because all my projects and everything I’ve planned are aimed at ensuring their stability.* 

(SP5 Male, 20 years old)

Well-being as a valuable direction was recognized by some of the participants:

*My well-being—that’s the most important thing for me.* 

(SP12 Male, 22 years old)

*Currently, it’s mainly my peace of mind, being able to improve my relationship with my parents, having a healthy relationship with them.* 

(SP4 Female, 19 years old)

Regarding health, one participant noted:

*My psychological and physical health; without health, there’s nothing.* 

(SP3 Female, 20 years old)

Others pointed to relationships as important aspects of their lives:

*My family and my friends.* 

(SP10 Female, 19 years old)

*My family, my friends, and my boyfriend.* 

(SP13 Female, 21 years old)

Professional development and achieving autonomy were also emphasized:

*Finishing my degree, that’s the most important thing.* 

(SP6 Male, 22 years old)

*My autonomy and my space are the most important things; [they are] the only things that depend on me.* 

(SP9 Female, 21 years old)

Regarding interferences that prevented the participants from focusing on what they truly desired, several actions were identified; these were aimed at satisfying external expectations or following persuasion by others.

*The things I do, I do them so that my mom feels proud of me. She doesn’t know that I’m homosexual and that makes many things difficult. But I want her to feel proud of me in other aspects, to see me as someone successful and to realize that my condition has nothing to do with what I can achieve.* 

(SP7 Male, 19 years old)

A significant number of the participants recognized that they struggled to keep their values in mind day-to-day, despite having identified them. When asked “*Have you ever forgotten about your values?,*” 9 of the 15 participants responded in the affirmative and explained what happened when they lost sight of them:

*I ended up really bad because I let my anger out.* 

(SP1 Male, 22 years old)

*Everything builds up, and I feel like sometimes I can’t handle it.* 

(SP12 Male, 22 years old)

Others described this situation as:

*I lose my direction.* 

(SP2 Male, 20 years old)

*Everything becomes chaotic.* 

(SP5 Male, 20 years old)

*Everything gets out of control.* 

(SP8 Female, 21 years old)

*I don’t make progress.* 

(SP10 Female, 19 years old)

Another type of interference that distanced the participants from their values included expressions of apathy, as reflected in a response to the question “*How would you like to be remembered?*”

*I wouldn’t like to be remembered. Everyone is free; I don’t do things to be admired or followed. I do things as I see fit. It sounds selfish, but I honestly don’t care because others are also responsible for themselves. Everyone has their own way of thinking and acting.* 

(SP9 Female, 21 years old)

With regard to the questions “*How do you see yourself in the future?*” and “*What things would you like to achieve?,*” their responses were often linked to fear of failure or uncertainty.

*It’s not something like “I want this or that in the future.” I don’t want to idealize my future because I don’t want to look back and realize I didn’t achieve what I wanted; that would affect me.*

These findings reveal a fragmented relationship with personal values, where clarity about what matters most coexists with difficulty in sustaining value-consistent action. Despite articulating meaningful life directions, many participants reported being distracted by external demands, unresolved emotional experiences, or internalized expectations, which disrupted their sense of coherence and progress. From the perspective of IPA, this disconnection highlights the existential tension between knowing what is important and being able to live accordingly underscoring the role of psychological flexibility in bridging intention and behavior, particularly in young adults navigating complex identity and social challenges.

#### 3.1.10. Manifestations Associated with Contacting Personal Values

Contacting personal values is an action that the study participants allowed themselves to carry out; most of them showed a willingness to explore what they wanted to be and achieve. This was clear in their responses to the question “*How would you like to be remembered?*”

*As the person who overcame himself, because my past was very dark. I was on the verge of collapse; I even distanced myself from my friends so they wouldn’t feel pain if something happened to me.* 

(Male, 22 years old)

*As a joyful, centered person who does everything with good intentions. I would like to be remembered as someone funny, capable of enjoying life.* 

(Male, 19 years old)

The same trend was observed in response to the question “*How do you see yourself in the future?*”

*Being the same cheerful and fun person, with many achieved goals, a good job, new friends, and people who surround me and bring good things into my life… Making my family happy.* 

(SP2 Male, 20 years old)

*Having overcome all these obstacles that stop and paralyze me, that don’t let me move forward.* 

(SP11 Female, 19 years old)

Likewise, the participants stated that they kept what was important to them present in their daily lives. Some of them commented:

*Every time I wake up, I try to remember what I want to achieve and that everything I have done so far has been for that.* 

(SP6 Male, 22 years old)

*I feel that we are a reflection of what we live. Many people tell me that I am loving because I grew up in a home like that; my dad is so loving with my mom, he respects her, loves her, is present, and is so responsible that I reflect that. So, I always keep that in mind in my life.* 

(SP13 Female, 21 years old)

The participants’ meaning making around their future and legacy revealed a dynamic process of identity construction rooted in personally significant values. Their reflections showed how aspirations, emotional experiences, and social learning contribute to shaping a coherent sense of self directed toward valued living. This process reflects a flexible engagement with values that guides purposeful action despite internal or external adversity, positioning value contact as a central component of adaptive functioning.

#### 3.1.11. Manifestations Associated with Behavioral Rigidity

Behavioral rigidity ranges from ineffective strategies to difficulty acting according to what individuals consider important. The participants who identified their values mobilized resources to achieve them but struggled to determine whether their actions were consistent with those values. When asked how they reached their goals, they focused their responses on concrete plans and steps rather than on their stated values. For example:

*I’m disciplined, so when I have clear goals, I give everything I have to achieve them—my effort, my time. I fully commit to it and, no matter what happens, I do it. I achieve it.* 

(SP2 Male, 20 years old)

*Well, first I organize my time so that the things I want to achieve can be done on time. Also, I surround myself with people who can help me achieve what I want.* 

(SP6 Male, 22 years old)

Some of the participants, although they had identified their values, had difficulty acting in accordance with them and attributed this to thoughts of failure and the emotional distress they may experience:

*I would love to travel, explore, put down roots somewhere else, but I worry that my grandmother might die, and I won’t be there.* 

(SP1 Male, 22 years old)

*I do everything short term. Long-term plans, like for a year from now, don’t work for me. I only act when I’m certain that I’ll get results.* 

(SP9 Female, 21 years old)

Other participants faced difficulties carrying out the actions necessary to fulfill their desires because of the obstacles they encountered, as shown in these examples:

*Well, it depends on the obstacle. If it depends on someone else, I’ll probably just set it aside and move on to something else. I have the ability to let go of things without [them] affecting me in the future.* 

(SP9 Female, 21 years old)

*It’s really hard for me—honestly, when something doesn’t go as I expected, thoughts of incompetence overwhelm me. That holds me back and stops me from continuing on the path I had already mapped out.* 

(SP11 Female, 19 years old)

A closer examination of the participants’ narratives revealed tensions between intention and action, illustrating a gap between identified values and committed behavior. This divergence frequently stemmed from internal obstacles such as anticipatory anxiety, fear of failure, or cognitive avoidance that limited sustained engagement with long-term goals. From an interpretative phenomenological perspective, such patterns could reflect experiential avoidance and diminished psychological flexibility, suggesting that while values may be clear, behavioral enactment often falters in the presence of emotional discomfort.

## 4. Discussion

This study offers a novel contribution by applying the Hexaflex model of ACT to explore psychological flexibility and inflexibility in university students through an interpretative phenomenological perspective. Unlike prior research that has relied primarily on quantitative approaches, this study provides a culturally grounded, in-depth understanding of how students navigate internal experiences and enact—or struggle to enact—their values in the face of academic, familial, and social pressures. These findings help bridge the gap between ACT’s theoretical constructs and the lived experiences of students in a Latin American higher education context, thereby broadening the empirical and cultural relevance of ACT-based research.

One of the most meaningful contributions of this study lies in illustrating how academic and cultural norms—such as “I must give everything,” “I have to achieve X,” or “family comes first”—shape students’ behavioral repertoires in rigid ways. Strategies like rumination, rationalization, or short-term decision-making, frequently described by participants, are not uncommon; in fact, they reflect patterns many of us engage in to some extent. However, these strategies may become problematic when they are reinforced by inflexible social rules that distance individuals from their own values. Our findings suggest that some students become caught between familial expectations and narrow definitions of academic success, leading to a disconnection from what truly matters to them. This highlights the potential value of interventions aimed at helping adolescents identify and discriminate their own values and align their actions accordingly—rather than merely following external rules that may not reflect their personal sense of a meaningful life.

### 4.1. Theoretical and Contextual Reflexivity

Throughout this study, the research team maintained a reflexive stance, acknowledging how our theoretical alignment with ACT and familiarity with psychological flexibility could influence both the interpretation of participants’ narratives and the thematic coding process. While this alignment enriched our sensitivity to subtle manifestations of (in)flexibility, we remained vigilant against imposing theoretical expectations onto the data. Instead, our iterative IPA process prioritized participants’ own words as primary evidence. This reflexivity underscores the recognition that our interpretations are co-constructed and inevitably shaped by our backgrounds as clinical psychologists familiar with both the cultural context and ACT principles. In the following sections, we discuss these results in relation to the existing literature.

#### 4.1.1. Experiential Avoidance vs. Acceptance

Our study revealed that the participants used control mechanisms to regulate their private events and perceived psychological distress negatively. Cognitive fusion with mental content, the search for justifications, and avoidance of aversive internal experiences predominated.

Wilson and Luciano described this as a vicious circle where attempts to control suffering result in increased distress [1]. The participants associated this struggle with exhaustion and frustration, which reflected the influence of their social and cultural contexts on their perceptions of suffering. The belief that pain must be avoided at all costs was reinforced, while well-being was mistakenly equated with happiness [1]. However, the participants did not mention the use of alcohol or drugs or other addictive behaviors as avoidance strategies. These behaviors, which alter internal events that generate distress, can lead to experiential avoidance disorder [2]. Our finding suggests that, for this group of students, substance use did not play a significant role in their emotional and cognitive regulation.

The participants’ narratives conveyed a lived tension between striving for emotional control and experiencing persistent discomfort. Rather than resolving their distress, these efforts appeared to reinforce a cycle of unease, which several students described as exhausting or emotionally draining. From their perspective, the pursuit of emotional suppression was not simply ineffective but deeply entangled with cultural ideals of strength and self-sufficiency.

#### 4.1.2. Self-As-Content vs. Self-As-Context

The participants tended to link their respective identities with self-criticism and the belief that their emotions and thoughts were inappropriate. They confused the socially constructed self with their continuous and timeless self, which hindered their ability to detach from the environment and attend to who they truly were. This attachment to verbal rules led them to strive for social conformity, which resulted in harsh and unrealistic self-evaluations [6].

Many of the participants associated their identity with external expectations, the need for parental approval, and the urgency to prove their worth to others, which limited their ability to act in alignment with their own values [6,11,12]. This self-critical stance emerged as the participants described a perceived gap between who they felt they ought to be and their current emotional experiences. Rather than expressing this explicitly in terms of social norms, their narratives conveyed a tension between personal authenticity and fear of disappointing others, often embedded in family dynamics or academic expectations. The lived sense of “not being enough” became a recurring thread, revealing how identity is shaped not only by internal scripts but also by unspoken emotional debts to significant others.

Another pattern observed was confusion between who they were and who they believed themselves to be based on past experiences. In this way, they constructed their self-definitions based on specific behaviors and events, thereby reinforcing the behavioral patterns that justified their self-perceptions.

These narratives expose an inner tension between the participants’ desire for authentic self-understanding and their tendency to cling to socially approved self-definitions. We recognize the possibility that, for some students, this strict self-scrutiny may serve protective or identity-cohering functions within their social context.

#### 4.1.3. Lack of Contact with the Present Moment vs. Contact with the Present Moment

The participants’ accounts revealed a recurring sense of disconnection from the present, often described through metaphors of automation and routine. Rather than articulating explicit avoidance, they conveyed an underlying sense of emotional detachment living life as a sequence of tasks rather than as a felt experience. This narrative thread suggests that productivity served not only as a cultural expectation but also as a coping scaffold, offering structure amidst inner chaos. A few participants, however, expressed subtle efforts to reclaim moments of presence, carving out spaces for connection and joy that briefly interrupted the rhythm of automatic functioning.

Modern life promotes the idea that well-being and success depend on productivity. Being constantly busy was a recurring pattern among the study participants—some were aware of it, while others were not. They lived on autopilot; they followed schedules and routines without paying attention to their thoughts or emotions and used distraction as an avoidance mechanism in response to loneliness, frustration, and psychological distress [11]. For them, this state seemed to serve an adaptive function by providing a false sense of progress, as they confused productivity with well-being while postponing connection with their inner world. Making endless to-do lists and feeling like there is never enough time reinforces the cultural belief that being idle is negative [14].

The students’ demonstrations reveal a paradox that permeates their daily lives: being constantly busy provides immediate relief from discomfort yet perpetuates a deeper undercurrent of emotional disconnection. While many students equated productivity with personal worth and a sense of progress, beneath this drive lies a subtle fear of confronting their own thoughts and feelings. The accounts reveal an ambivalence between valuing routine as a source of order and recognizing how it can become a barrier to genuine contact with the present moment. Notably, a few participants hinted at moments of awareness and resistance, suggesting that even within rigid schedules, there is a latent desire for more mindful and fulfilling engagement with life.

#### 4.1.4. Fusion vs. Defusion

The need to make sense of psychological distress drives individuals to organize their thoughts logically, thereby leading to a fusion between verbal/cognitive processing and direct experience, which makes differentiation difficult [8]. Among the study participants, this tendency manifested in three ways: the students became entangled in distressing thoughts or emotions, struggled to gain perspective, or allowed past experiences to interfere with the present. Although these strategies were intended to create coherence, the students ended up rigidly regulating their behavior without taking additional information into account [8].

This pattern was vividly expressed in the narratives through metaphors of “getting stuck” or “looping” in their minds. The participants frequently described being unable to stop thinking, despite recognizing that doing so led to emotional exhaustion. One student explained that “my thoughts go round and round like a wheel I can’t get off,” while another noted that “remembering things that happened before takes over everything, and I can’t react to what’s happening now.” These descriptions capture the lived experience of fusion—not merely as a cognitive event but as a felt struggle to separate thought from action and past from present.

In this regard, Blackledge [10] argued that people believe in their thoughts so strongly that they take them as reality itself, which affects their behavior and interpersonal relationships and reinforces inflexible behavioral patterns.

Defusion requires training and a willingness to change one’s relationship with one’s thoughts rather than eliminating them [8]. Some of the participants recognized fusion as exhausting and draining, as it involves trying to control the uncontrollable. As Blackledge [10] pointed out, thoughts can become problematic when one cannot determine when to consider them and when to let them go.

This narrative rigidity coexists with fleeting moments of awareness that thoughts do not have to define reality, highlighting an underlying tension between the comfort of familiar mental scripts and the challenge of adopting a broader perspective. The few instances of defusion suggest that distancing oneself from one’s own mental content is experienced as liberating yet remains an unfamiliar and uncertain process, requiring a shift from habitual control strategies to more flexible and accepting stances.

#### 4.1.5. Lack of Contact with Values vs. Contact with Values 

The study participants were able to identify areas that gave meaning to their lives. Family was most frequently mentioned, and it was associated with notions such as totality (“my family is everything to me”), motivation (“she is my driving force”), reward (“I want to give that achievement to my parents”), hierarchy (“family should always come first”), and support (“my mom is unconditional”). They also emphasized education as a means to achieve goals, mental health, friendship, and a sense of self-linked to peace and autonomy. Only one participant mentioned romantic relationships.

Although the participants appeared to have clarity about their values, we identified interferences to their genuine connections. One such interference was the tendency to satisfy external expectations. In this regard, Hayes [6] warned that confusing immediate gratification with life meaning leads to actions motivated by fear of disapproval and a disregard for personal motivations that bring well-being.

This disconnect was expressed in participants’ narratives as a felt tension between personal aspiration and social approval. While their verbal responses suggested clarity, the tone and hesitation observed in interviews indicated uncertainty. Some participants spoke of values as ideals they “should” follow rather than intrinsic commitments. The presence of conditional phrasing (“I guess it matters,” “they expect that from me”) hinted at a fragile connection between values and self-concept. These accounts point not only to a cognitive awareness of what matters, but also to the emotional ambivalence that undermines its enactment.

Another manifestation of a lack of connection with values was indifference toward life’s purpose. Wilson and Luciano [1] explained that such responses act as protective mechanisms against fear of failure or rejection.

In ACT, values are the ongoing choices that guide life and are unlike goals, which are finite [1]. Keeping values present helps prevent social control and avoid discomfort taking over [8]. When the study participants lost sight of their values, they described their experiences using terms like loss, lack of control, and directionlessness: “I lose my way,” “everything gets out of control,” and “I’m not making progress.”

In sum, while the participants were able to articulate their values, engaging with them in a sustained and meaningful way proved difficult. Moments of detachment or indifference did not reflect an absence of values, but rather pointed to protective strategies—ways of avoiding the emotional risks tied to failure, disappointment, or disapproval. This highlights how internalized fears and social pressures can blur the distinction between authentic choice and rule-driven behavior, making values-based action a fragile and often elusive process.

#### 4.1.6. Inaction vs. Committed Action

Psychological inflexibility sustains distress by leading individuals to avoid situations that might involve failure or discomfort [15]. Among the participants, this was often reflected in a difficulty in recognizing when their actions were misaligned with their values. Instead of acting from guiding principles—such as family, well-being, or education—their decisions were often driven by external expectations or short-term, concrete goals. As Hayes [8] suggests, fusion with negative thoughts narrows one’s ability to consider alternative paths and act in line with personal values.

The participants frequently described their decisions as things that “had to be done,” rather than as choices aligned with what truly mattered to them. This disconnect generated unease, as captured by expressions like “doing things because I should, not because I want to,” or retreating altogether: “If I’m not sure I’ll succeed, I don’t even try.” These accounts illustrate how avoidance is not merely passivity, but a protective strategy shaped by fear of failure, perfectionism, and fear of disappointing others. Through the IPA lens, such behaviors reveal an internal struggle with vulnerability and perceived inadequacy.

Emotional avoidance also emerged as a defense mechanism, particularly in relation to interpersonal relationships and self-expectations. Attempts to suppress discomfort often reinforced suffering and deepened disconnection from personal values. This pattern aligns with the idea that avoidance and fusion jointly obstruct committed action [8].

Committing to value-based action requires flexibility—the willingness to move forward even when outcomes are uncertain or emotionally challenging. As Hayes [6] notes, acting from personal values, rather than external demands, enables a more authentic and fulfilling life. This process demands self-awareness and the courage to confront discomfort, allowing individuals to break free from self-imposed limitations.

This theme reveals that inaction among students was not a lack of motivation, but a fragile effort to maintain control and avoid emotional exposure. Their narratives reflected a tension between the desire for progress and the fear of failure, resulting in rigid routines and short-term strategies. Even moments of committed action appeared contingent on certainty and validation, suggesting that sustained value-driven behavior remains difficult when dominated by avoidance and internalized pressure.

##### Reflexivity, Strengths, and Limitations

This study possesses notable strengths that enhance its contribution to the understanding of psychological flexibility and inflexibility among university students. By employing semi-structured interviews, this research provided an opportunity to explore dimensions of the participants’ internal experiences that are often overlooked by self-report questionnaires. Many students emphasized that this was the first time they had spoken openly about their emotional experiences within the academic context, highlighting the importance of being listened to without judgment. Several participants described the interview as a safe space that allowed them to express and process feelings they had not previously acknowledged, thus serving as a valuable moment of self-awareness and reflection.

Regarding limitations, it is recognized that the sample was drawn from a single university, which may limit the generalizability of the findings. However, the University of Magdalena has an enrollment of over 24,000 students across 32 different academic programs, ensuring a degree of internal diversity. Rather than claiming statistical representativeness, this study prioritizes the transferability of its insights to similar higher education contexts. Social desirability bias and cultural norms related to emotional expressiveness could have influenced the narratives, yet the aim was not to guide or constrain participants’ responses but to explore how they naturally describe their experiences of flexibility and inflexibility in everyday life.

These findings reaffirm the relevance of listening deeply to students’ experiences as a means of understanding how they navigate internal conflicts, value tensions, and external pressures. Beyond theoretical contributions, this study demonstrates that making space for students’ voices is essential to uncovering the complexity of psychological (in)flexibility in context. The narratives collected here underscore the importance of recognizing students not merely as data points, but as meaning-makers whose lived experiences can guide the development of more responsive, context-sensitive interventions in higher education settings.

## 5. Conclusions

In terms of mental health, university students have been identified as a vulnerable population [28]. Within the educational context, a negative self-perception can significantly affect academic performance by causing psychological distress and making it difficult for students to carry out the academic and social activities inherent to university life.

This study advances that understanding by revealing how, in a Latin American university context, students’ struggles with internal experiences are maintained by deep-seated cultural scripts of stoicism, family-driven approval, and an overvaluation of productivity as self-worth. These cultural narratives seem to function as verbal rules, the adherence to which influences the processes described in the Hexaflex model in various ways. For example, the expectation to suppress emotions in the name of endurance often reinforces experiential avoidance, while strong family obligations may lead students to pursue externally imposed goals, disconnecting them from personally chosen values. Similarly, the internalization of productivity as a measure of self-worth can increase cognitive fusion with self-critical thoughts, which, in turn, fosters ruminative patterns driven by cultural pressures to control distress through persistent mental analysis. This process not only undermines present-moment awareness and committed action but may also lead students to live a life guided by external demands rather than by their own values, ultimately disconnecting them from what truly matters to them. Instead of promoting flexible engagement with challenges, these cultural pressures may contribute to rigid behavioral patterns aimed at maintaining social approval or avoiding perceived inadequacy. In this way, the cultural context shapes how students relate to their thoughts, emotions, and goals—often reinforcing inflexibility to the detriment of well-being and personal growth.

Theoretically, these findings reinforce and expand ACT principles by situating them within a cultural frame rarely addressed in the existing literature. They highlight how universal processes of psychological inflexibility are locally configured by familial expectations and social beliefs about success and resilience. This context-sensitive perspective broadens the cross-cultural validity of the ACT model and demonstrates its applicability beyond Western settings. In practice, these insights argue for a paradigm shift in how universities approach student mental health. Rather than focusing solely on symptom alleviation, interventions should target the development of psychological flexibility as a protective factor for well-being and academic success. Universities could integrate Hexaflex process-informed workshops into curricula, train staff to recognize inflexibility patterns, and create safe relational spaces where students can reflect on their identities without fear of judgment. Recognizing and legitimizing students’ unspoken emotional burdens could bridge a critical gap in student support services. These recommendations align with recent controlled trials and mixed-method studies demonstrating that brief ACT-based programs and online interventions can strengthen students’ stress tolerance, self-regulation, and study skills [18,20,21].

Future research should expand this work by comparing students from varied cultural and institutional contexts, testing how cultural values interact with Hexaflex processes, and employing longitudinal designs to examine how psychological flexibility evolves with targeted interventions and life transitions. Combining qualitative depth with quantitative measures may also yield a more comprehensive understanding of protective and risk factors.

In conclusion, this study offers critical insights into how university students navigate the tension between cultural expectations and their internal experiences, revealing patterns of avoidance and rigid self-concepts that can limit both personal growth and academic performance. By situating these psychological processes within a Latin American university context, this research highlights the need for culturally sensitive interventions that promote psychological flexibility as a protective resource for students’ well-being and success. Notably, these findings contribute to extending the ACT framework beyond Western-centric assumptions, demonstrating its cross-cultural applicability through evidence drawn from Latin American contexts. Future research should build on this evidence by testing culturally tailored ACT protocols in diverse Latin American universities, expanding on the promising results observed in Europe and Asia [18,22], supporting the development of evidence-based practices that empower students to live in alignment with their authentic values, even amidst social and academic pressures.

## Figures and Tables

**Table 1:** Demographic and academic characteristics of the participant sample.

Code	Sex	Age	Academic Program
SP1	Male	22	Agronomy Engineering
SP2	Male	20	Public Accounting
SP3	Female	20	Medicine
SP4	Female	19	Biology
SP5	Male	20	Law
SP6	Male	22	Nursing
SP7	Male	19	Public Accounting
SP8	Female	21	Nursing
SP9	Female	21	History and Heritage
SP10	Female	19	Nursing
SP11	Female	19	Business Administration
SP12	Male	22	Systems Engineering
SP13	Female	21	Industrial Engineering
SP14	Male	18	International Business
SP15	Female	19	Public Accounting

**Table 2:** Steps for IPA data analysis.

Steps	Description
Initial familiarization with a case and initial comments	Reviewing interviews and notes
Initial identification of themes	Detailed descriptive, linguistic, and conceptual comments
Looking for connections between themes	Identifying key themes for each case
Producing a table of themes	Describing and illustrating emerging themes
Continuing with further cases	Repeat idiographic analyses, identifying shared and divergent patterns
Writing up the analysis	Write the results of the analysis

Note: adapted from *Introduction to Research Methods in Psychology* by D. Howitt and D. Cramer, 2011, Book Title, Pearson Education Limited, p. 391 [32].

**Table 3:** Mapping ACT processes, themes, and participant quotes.

ACT Process	Superordinate Theme	Emergent Theme	Illustrative Quotes (Participant)
**Experiential Avoidance** (suppressing discomfort)/**Acceptance** (willingness to experience discomfort)	Control of private events	Tendency to focus on private events, disrupting contact with the present (fusion) - Search for reasons to explain emotional experiences - Tendency to invalidate emotions, redirecting attention - Avoidance patterns (withdrawal, confrontation avoidance)	I think about it daily, like 15–16 h a day (SP1)
	Acceptance of private events	Willingness to admit thoughts/emotions perceived as aversive, without futile control	If I see I can’t solve it, I just let it go and move on. I feel lighter (SP6)
**Self-as-Context** (observes self beyond thoughts)/**Self-as-Content** (identifies self with thoughts)	Defined by thoughts	Self-criticism for having certain thoughts or feelings - Belief that thoughts/emotions are bad or wrong - Tendency to equate self with thoughts, shaped by context	I know I have anger issues… so I avoid getting angry (SP1)
	Observing self without judgment	Ability to maintain perspective beyond negative thoughts/emotions	I’m still afraid of many things, but I keep going despite the fear (SP2)
**Lack of Contact with the Present** (disconnected and caught in past or future)/**Present Moment Contact** (open and engaged with present moment)	Living on autopilot	Doing tasks non-stop, moving from one to another without noticing feelings/thoughts	I do so many things I don’t have time to feel bad (SP10)
	Mindful presence	Openness to contact what is felt/thought in the here and now	I have a routine, but I enjoy my day; I don’t get lost in it (SP6)
**Cognitive Fusion** (takes thoughts as literal truth)/**Defusion** (sees thoughts as thoughts)	Fusion with private content	Ineffective perspective-taking, over-identifying with thoughts - Past emotions interfering with present experience - Hooked on distressing thoughts or feelings	I made up movies in my head that he was cheating (SP7)
	Defusion from private content	Recognizing internal events as ideas, possibilities, not fixed reality	I learned to be a spectator, especially when I can’t control it (SP13)
**Values** (has direction and meaning)/**Lack of Values** (lacks direction or meaning)	Difficulty contacting values	Acting to meet others’ expectations - Difficulty keeping values in mind day-to-day - Apathy or statements of detachment	I do things so my mom feels proud. She doesn’t know I’m homosexual (SP7)
	Contact with values	Openness to explore what really matters and give life meaning - Commitment to keep values present	I would like to be remembered as someone funny, capable of enjoying life (SP13)
**Inaction** (avoids action due to discomfort)/**Committed Action** (takes value-based action)	Behavioral rigidity	Ineffective strategies to implement actions - Inaction due to fear of failure or worries - Stories of past failure or uncertainty about future success - Difficulty aligning actions with values	I do everything short-term; I can’t plan long-term (SP9)
	Values above all	Commitment to values-driven actions despite difficulty	I give everything to reach my goals; I fully commit until I get it (SP2)

Note: table compiled by the first author.

## Data Availability

The data that supports this research is available and can be requested by email at wcervantes@unimagdalena.edu.co.

## Abbreviations

ACT	Acceptance and Commitment Therapy
IPA	Interpretative Phenomenological Analysis

## Appendix A

This semi-structured interview guide was developed to explore college students’ understanding and experience of psychological flexibility and inflexibility, drawing on the six core processes of the ACT Hexaflex model. The guide includes introductory questions, process-specific prompts, and closing reflections. The interview was designed to elicit rich, first-person accounts of how participants interpret their internal experiences and behavioral strategies. Given the broader scope of the full guide, only a selection of representative questions is presented below. Additional or adapted questions were used as needed to deepen the exploration based on participants’ responses.

**Table A1:** Semi-structured interview guide.

Section	Purpose	Sample Questions
Introduction	Introduce the study and explain the process	- This study is part of a doctoral research project at the Autonomous University of Madrid, whose objective is to explore psychological flexibility and inflexibility in university students. - There are no right or wrong answers. The interview is confidential and will be audio recorded for transcription purposes only.
Personal and Family Context	To contextualize the participant	- How old are you? - What academic program are you currently studying? - Who do you live with? - How would you describe your relationship with them?
Perceptions of Psychological Flexibility	Explore participants’ understanding of the concept	- Have you heard of psychological flexibility before? - What do you think people your age understand by this term? - And what do you understand by it? Can you give an example? - What traits would a psychologically flexible person have? - If it were a recipe, what ingredients would it need?
Psychological flexibility processes	Elicit personal strategies aligned with ACT’s six core processes	**Acceptance** - When you feel bad, do you try to control it or allow yourself to feel it? - What happens when you accept those feelings? **Self-as-Context** - How do you define yourself? - Does this self-view influence your behavior? - What would help you change negative self-concepts? **Defusion** - Have you ever believed a “story in your head”? - How did you realize it wasn’t real? **Present-Moment Awareness** - Do you feel like you live on autopilot? - How do you bring yourself back to the present? **Values** - What things are most important to you? - Do you live according to your values? **Committed Action** - What actions do you take toward what matters? - What gets in the way, and how do you respond?
Closing	Emotional closure and ethical care	- How do you feel after this conversation? - Is there anything else you’d like to add or ask? - Thank you for your time. Your responses are valuable for understanding how students experience psychological flexibility.
